# Supplementary material for: Thermal and Mechanical Characterization of Epoxy/Polyimide Blends via Postcuring Process
Source: Polymers (Basel). 2023 Feb 21;15(5):1072. doi: 10.3390/polym15051072 (PMC10007675; doi:10.3390/polym15051072)
Supplement: Supplementary file 1 [file polymers-15-01072-s001.zip › polymers-2190324-supplementary.pdf]

Supplementary Information

Thermal and Mechanical Characterization of Epoxy/Polyimide Blends via Post-Curing Process

Yong-Min Lee[1], Kwan-Woo Kim[1,†], Byung-Joo Kim[2,†]

[1]Convergence Research Division, Korea Carbon Industry Promotion Agency, Jeonju 54852, Republic of Korea

[2]Department of Carbon-nanomaterials Engineering, Jeonju University, Jeonju 55069, Republic of Korea

Correspondence: kkw1988@kcarbon.or.kr; Tel.: +82 63 219 3713

Correspondence: kimbyungjoo@jj.ac.kr; Tel.: +82 63 220 3293

Supporting table captions

Table S1. Nomenclature of Epoxy/Polyimide Blends Mixed with Stoichiometric Equivalent Ratio Equation

Table S2. T_{ci}, T_{cp}, T_{cf}, and ΔT_{cif} Values of Epoxy/Polyimide Blends with Various Polyimide Contents

Supporting Figure Captions

Figure S1. Chemical structures of epoxy, polyimide, and 4,4′-diaminodiphenylmethane.

Figure S2. Thermogravimetric analysis (TGA) thermograms of polyimide and NMP analyzed in N_2 gas atmosphere.

Figure S3. A schematic showing curing mechanisms of epoxy/polyimide and epoxy/DDM reaction.

Figure S4. Schematic representation of (a) epoxy/DDM and (b) epoxy/polyimide cross-linking network.

Figure S5. Schematic representation of S1, S2, and S3 for A* and K*.

Table S1. Nomenclature of Epoxy/Polyimide Blends According to Stoichiometric Equivalent Ratio Equation

Nomenclature	Epoxy (g)	DDM (g)	Polyimide (g)	Curing time (h)	Post curing time (h)
EP	100	26.09	0	1	-
EPI-1		24.41	10		
EPI-2		22.72	20		
EPI-3	100	21.04	30	1	-
EPI-4		19.35	40		
EPI-5		17.67	50		
EPI-5-1					1
EPI-5-5	100	17.67	50	1	5
EPI-5-10					10

Table S2. T_{ci}, T_{cp}, T_{cf}, and ΔT_{cif} Values of Epoxy/Polyimide Blends with Various Polyimide Contents

**Sample**	**T_{ci} (°C)**	**T_{cp} (°C)**	**T_{cf} (°C)**	**ΔT_{cif} (°C)**
EP	126.20	168.05	205.77	79.57
EPI-1	116.70	166.32	204.58	87.88
EPI-2	114.87	162.39	218.94	104.07
EPI-3	101.68	163.64	231.80	130.12
EPI-4	105.51	165.18	237.23	131.72
EPI-5	103.25	167.62	239.01	135.76

T_{ci}: Initial curing temperature

T_{cp}: Curing peak temperature

T_{cf}: Curing finish temperature

ΔT_{cif}: Range of curing temperature

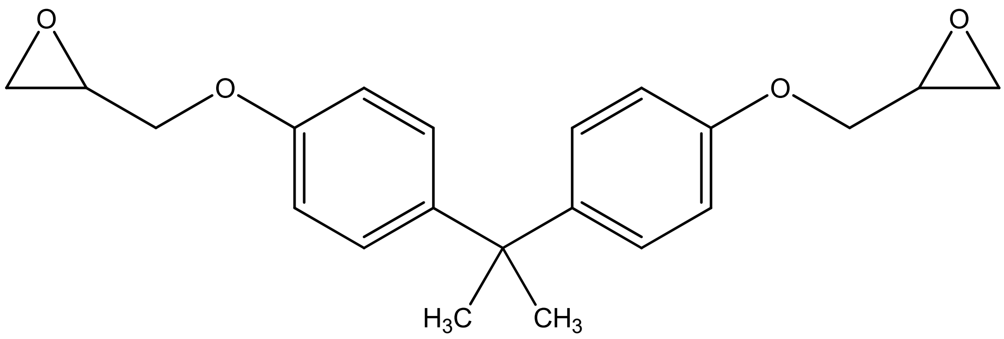

Epoxy resin (DGEBA)

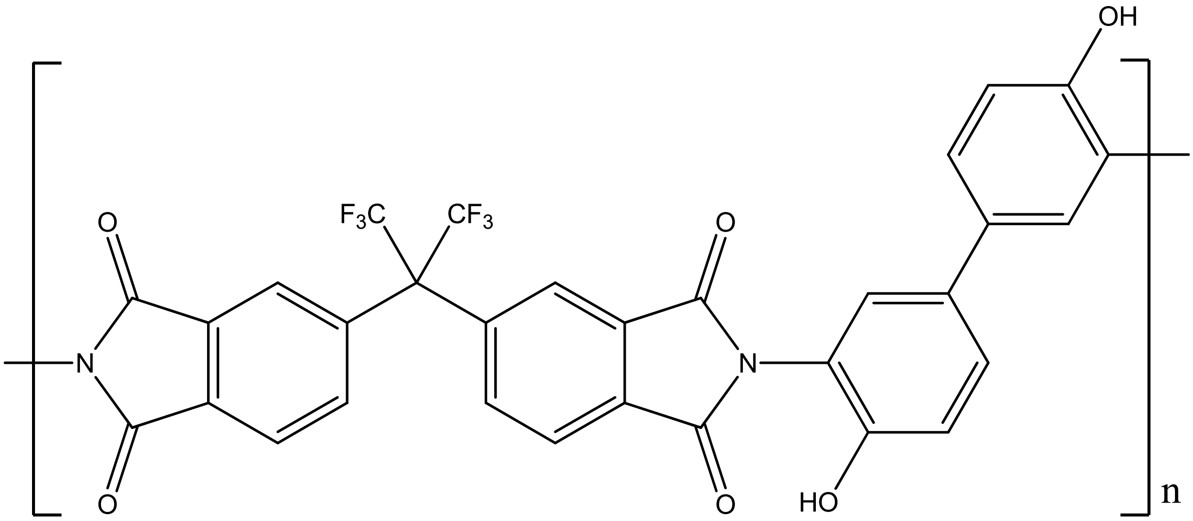

Polyimide

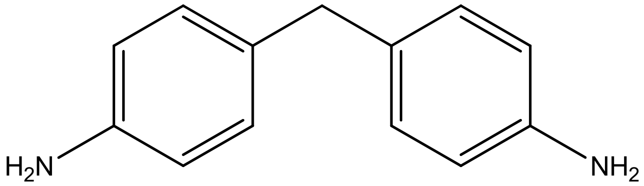

4,4'-Diaminodiphenylmethane

Figure S1. Chemical structures of epoxy, polyimide, and 4,4′-diaminodiphenylmethane.

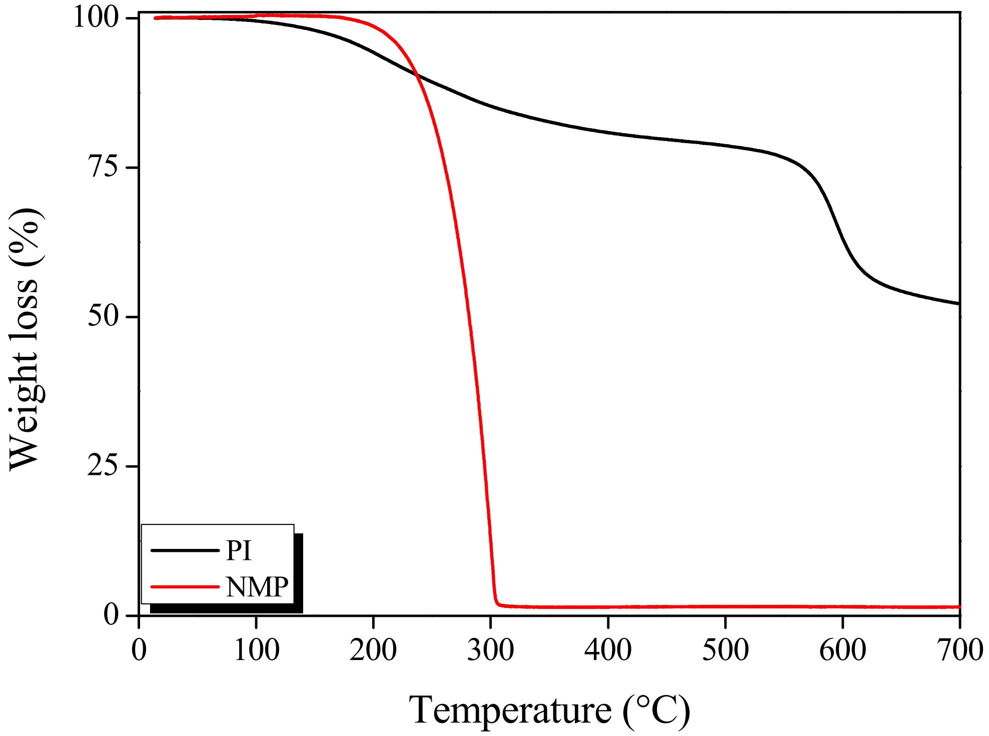

Figure S2. Thermogravimetric analysis (TGA) thermograms of polyimide and NMP analyzed in N_2 gas atmosphere.

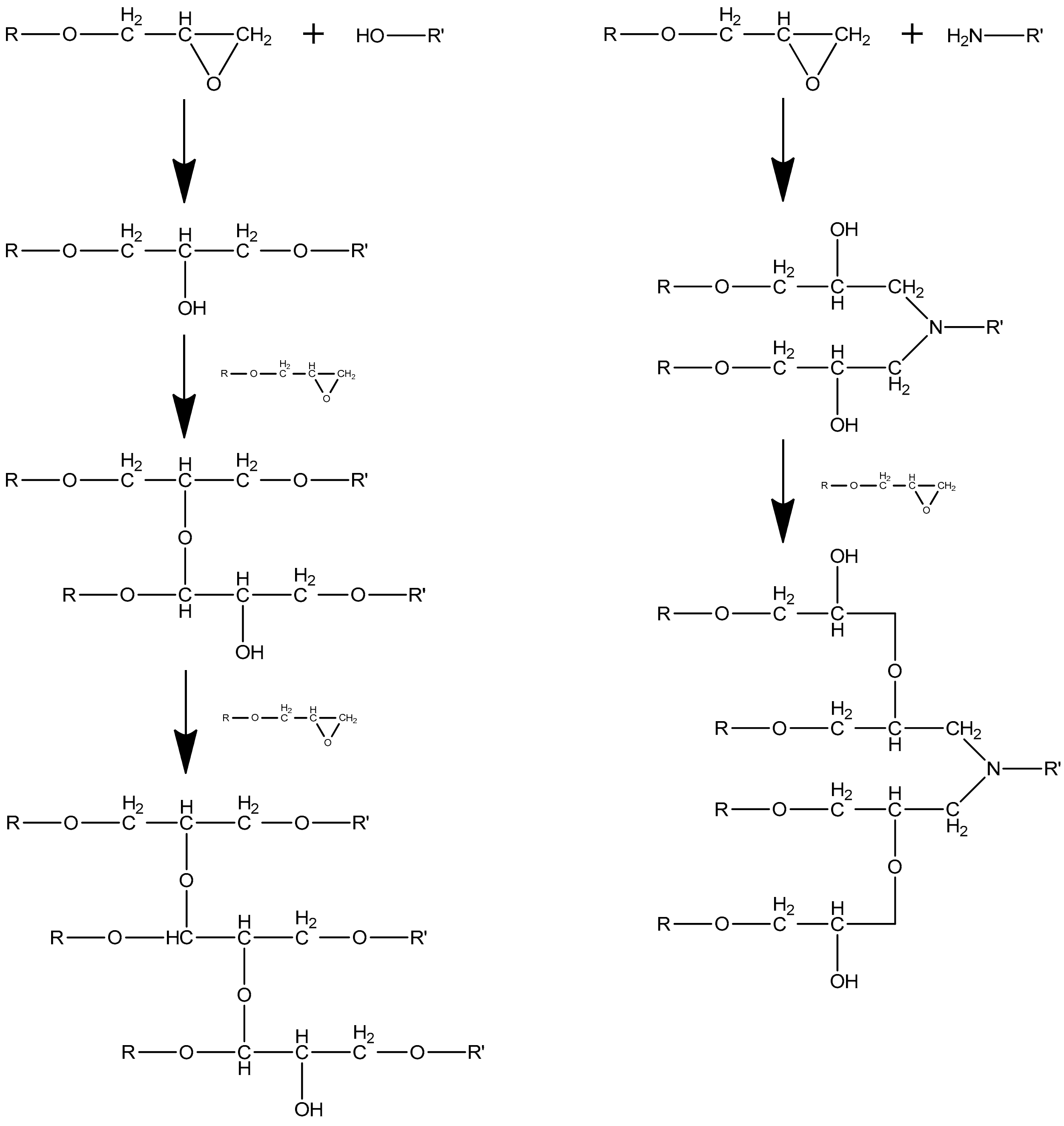

Figure S3. A schematic showing curing mechanisms of epoxy/polyimide and epoxy/DDM reaction.

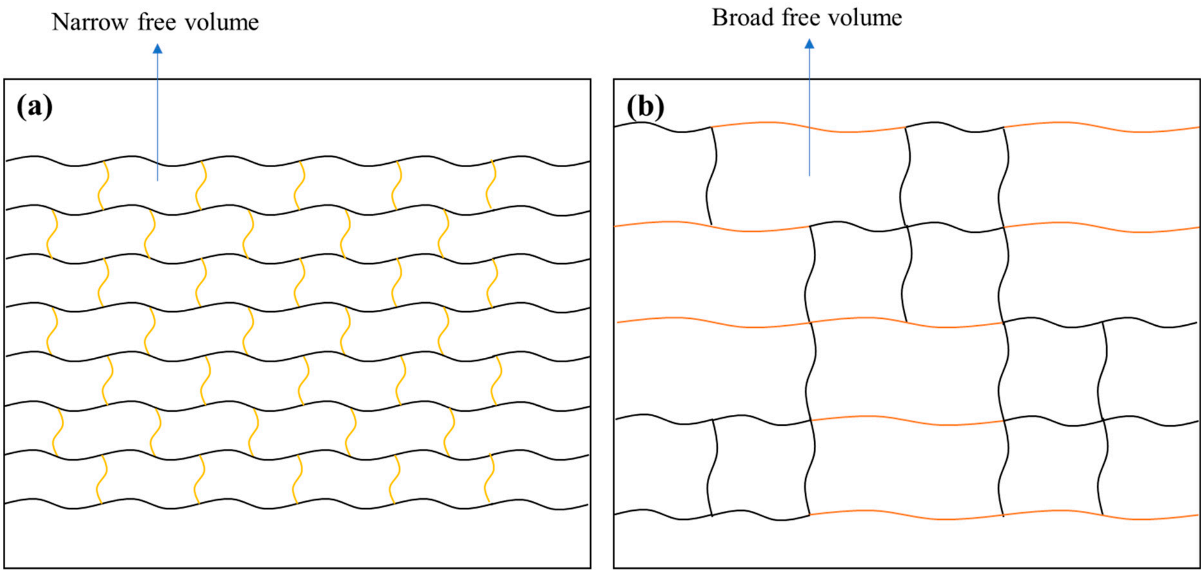

Figure S4. Schematic representation of (a) epoxy/DDM and (b) epoxy/polyimide cross-linking network.

*Black line: epoxy

**Yellow line: DDM

***Orange line: polyimide

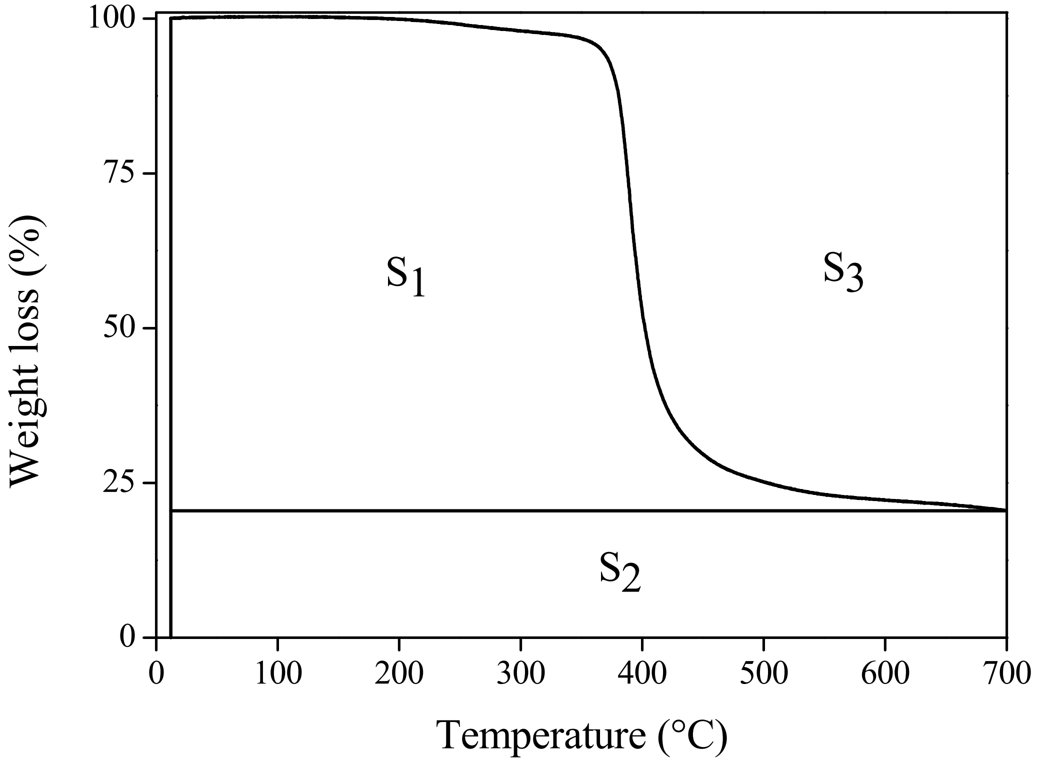

Figure S5. Schematic representation of S_1, S_2, and S_3 for A* and K*.
